# Progression of local kyphosis after conservative treatment for compressive cervical spine fracture with spinal cord injury

**DOI:** 10.1186/s13018-019-1115-z

**Published:** 2019-04-11

**Authors:** Kazuya Yokota, Takeshi Maeda, Osamu Kawano, Eiji Mori, Tsuneaki Takao, Hiroaki Sakai, Muneaki Masuda, Yuichiro Morishita, Tetsuo Hayashi, Kensuke Kubota, Yasuharu Nakashima

**Affiliations:** 1 Department of Orthopaedic Surgery, Japan Labor Health and Welfare Organization, Spinal Injuries Center, 550-4 Igisu, Iizuka, Fukuoka, 820-0053 Japan; 2 Departments of Orthopaedic Surgery, Graduate School of Medical Sciences, Kyushu University, 3-1-1 Maidashi, Higashiku, Fukuoka, 812-8582 Japan; 3 Present Address: Division of Genetics and Development, Krembil Research Institute, University Health Network, 60 Leonard Avenue, Toronto, ON M5T2S8 Canada

**Keywords:** Cervical spinal cord injury, Local kyphosis, Vertebral fracture, Laminar fracture

## Abstract

**Introduction:**

Compressive-flexion type cervical spine fracture is typically accompanied by apparent dislocation of the facet joints, undesirable cervical alignment, and devastating neurological dysfunction, which provides strong rationale for rendering prompt operative treatment. However, the validity of conservative treatment for compressive-flexion cervical spine injury in cases with preserved congruity of the facet joints has yet to be elucidated. The purpose of this study is to evaluate the long-term outcome of cervical alignment following conservative treatment for compressive-flexion cervical spine injury with preserved congruity of the facet joints.

**Methods:**

A total of 662 patients who experienced spinal cord injury from 2007 to 2017 were included and underwent retrospective review in a single institute. Thirteen patients were identified as receiving conservative therapy following compressive-flexion cervical spine fractures with spinal cord injury. Clinical and radiological results were collected, including vertical fractures of the vertebral column, laminar fractures, progression of local kyphosis, and neurological status. The degree of the local cervical kyphosis was evaluated with two methods: the posterior tangent method and the endplate method.

**Results:**

All 13 patients were male, and the mean age at the time of injury was 28.4 years. The mean follow-up period was 3 years. Although none of the patients presented neurological deterioration after the injury, the degree of local kyphosis was increased at the time of final follow-up compared to what was observed at the time of injury. Patient age at the time of injury and concurrent vertical fracture of vertebral body could have been influencing factors for the progression of the kyphosis. While laminar fracture affected the kyphosis at the time of injury, it was not a strong influencing factor of the overall progression of local kyphosis.

**Conclusions:**

The conservative option for the compressive-flexion cervical injury allowed us to treat without exacerbating neurological symptoms as long as the facet joints are preserved. However, in terms of cervical alignment, surgical stabilization may have been desirable for these patients. Notably, the younger patients and the patients with vertical fracture of the cervical vertebral column in this type of injury required closer observation to help prevent the progression of local kyphosis.

## Key points

- We reported the progression of local kyphosis following conservative treatment for compressive-flexion cervical spine fractures with spinal cord injury.
- Younger patients with such fractures had a higher progression of local kyphosis following injury.
- The patients who had vertical vertebral fractures were also associated with higher progression of local kyphosis compared to those without vertebral vertical fractures.
- Laminar fracture affected the initial kyphotic alignment of the cervical spine at the time of injury, but the laminar fracture was not an influencing factor for the long-term progression of local kyphosis.

## Mini abstract

We report on 13 patients to present the progression of cervical kyphosis after conservative treatment for compressive-flexion cervical spine fractures concurrent with spinal cord injury. Younger patients and patients with vertical vertebral fractures demonstrated higher incidence of progression of local cervical kyphosis.

## Introduction

Cervical spine fracture is caused by a traumatic event, such as a motor vehicle accident or a fall [1]. It is categorized into several types, based on the mechanism of the injury [2, 3]. Compressive-flexion fracture is one such type of spinal injury [4], which causes severe damage directly to the spinal cord as well as to spinal bony structures [5]. Patients with cervical spinal cord injury suffer from permanent motor/sensory dysfunction and mental/economic burdens following injury [6, 7]. In addition to these issues, these patients incidentally suffer from progression of spinal deformity, such as kyphosis and scoliosis [8]. However, the risk factors for progression of spinal deformity have yet to be elucidated.

The desirable treatment policy for compressive-flexion cervical fracture with spinal cord injury is a surgical approach including fixation, decompression, and correction of the cervical alignment [9]. Previous reports have shown that fixation and decompression after cervical spinal cord injury improved functional recovery and prevented the deterioration of neurological findings [10, 11]. Recent development of the subaxial injury classification (SLIC) scoring system recommends the surgical approach for patients with cervical spine fractures and incomplete/complete paralysis brought about by spinal cord injury [12, 13]. However, some patients with compressive-type fracture maintain the congruity of the facet joints and present acceptable cervical alignment despite concurrent spine fracture and spinal cord injury [5]. Furthermore, these patients possibly suffer from concomitant multiple trauma and/or hemorrhagic shock after injury [14, 15], which often provokes reluctance to pursue surgery due to its invasiveness and potential risk of further complications [16]. As a result, conservative treatment for cervical spinal fracture with spinal cord injury remains an attractive option. To the best of our knowledge, however, few reports exist regarding the long-term outcomes of conservative treatment for patients with compressive-flexion cervical fracture with spinal cord injury.

We retrospectively analyzed the clinical characteristics, radiological findings, and treatment outcomes of 13 patients who suffered cervical spinal fracture with spinal cord injury and who were conservatively treated in our department. The objective of the present investigation was to report long-term outcomes in these patients and to explore the potential risk of progression of local kyphosis following injury.

## Materials and methods

**Fig. 1:**
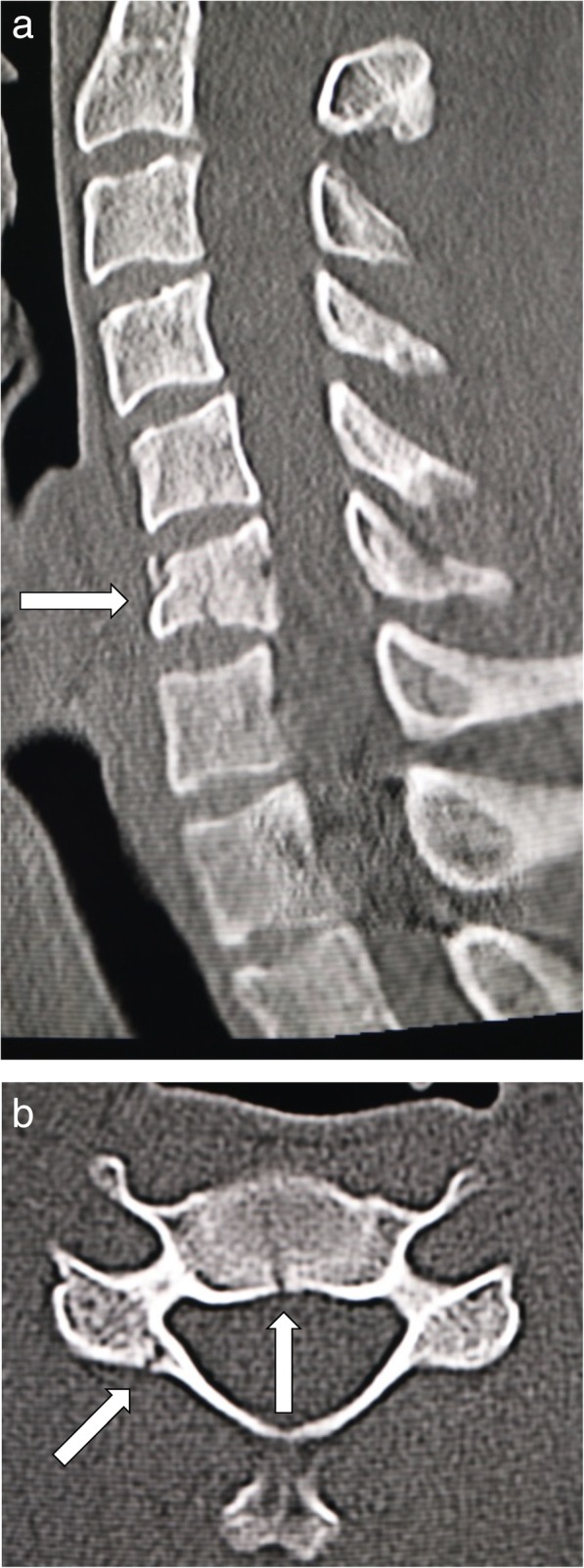
A radiographic example of a patient included in this study. **a** The patient suffered from compressive-flexion vertebral fracture with preserved cervical alignment. **b** The vertical fracture of the vertebra and the right laminar fracture occurred concurrently in this patient

The database of spinal cord injuries at the Spinal Injuries Center in Fukuoka, Japan, was researched for all cervical spine injury cases occurring between 2007 and 2017. A retrospective chart and radiological image review was performed to examine radiographic features, neurological findings, and treatment results. All patients underwent plain radiographs plus computed tomography (CT) or magnetic resonance imaging (MRI) for diagnosis. Compressive-flexion cervical spine injury was diagnosed with plain lateral radiographs, CT, and MRI when an apparent fracture of the vertebral body was observed, especially with evident loss of height at the anterior part of the body. The integrity and congruity of the facet joints were confirmed with CT and MRI images [17]. All patients had evidence of spinal cord injury, as represented by a signal change in the injured spinal cord in T2-weighted MRI images. Representative images of compressive-flexion injury are shown in Fig. 1. Patient age, sex, follow-up period, mechanism of the injury, level of the injury, vertical fracture of the vertebral body, laminar fracture, type of orthosis, and neurological findings were analyzed. The results of the neurological analysis were classified according to the American Spinal Injury Association (ASIA) Impairment Scale, which is an international standard for neurological classification of spinal cord injury [18].

### Statistical analysis

The Wilcoxon rank sum test was used to compare the median values of the quantitative data for cervical kyphosis as measured by the posterior tangent method and the endplate method. Correlational analyses were performed using the Pearson correlation coefficient. In all statistical analyses, significance was defined as *P* < 0.05. The values for the groups are presented as the average ± standard error of the mean (SEM). All statistical analyses were carried out using the JMP software program (version 13; SAS Institute).

## Results

During the 11 years between 2007 and 2017, 662 patients with cervical spinal cord injury were treated at our single facility. Of these, we found 13 patients (2%) who received conservative treatment, such as stabilization of the cervical spine by rigid orthosis via neck brace or halo vest with intensive rehabilitation. The frequency of patients treated conservatively was quite low because most of the patients with both spinal cord injury and spinal fracture were referred for treatment with a surgical approach [12]. Most of the included patients were forced to choose conservative treatment due to complications in other organs, such as brain subarachnoid hemorrhage, brain subdural hygroma, liver injury, spleen injury, or multiple fractures in the limbs. We also intentionally chose conservative treatment for some of the patients due to their maintained cervical alignment after injury. These included 13 male patients between the ages of 14 and 68 years of age (mean age 28.4 years). A summary of clinical findings is shown in Table 1. The follow-up period was between 9 months and 9.5 years (mean period 3 years). At the time of final follow-up, bony fusion was observed in all patients. Additionally, in terms of the neurological findings, one patient was classified as ASIA Impairment Scale (AIS) grade A, three patients as grade B, two patients as grade C, and seven patients as grade D at the time of injury (Table 2). None of these patients’ neurological statuses was exacerbated following conservative treatment as observed at the time of final follow-up (Table 3).

**Table 1:** Patient demographics of this study

Case	Age (yr)/sex	Follow-up period (days)	Mechanism of injury	Level of injury	Vertical fracture	Laminar fracture	Orthosis for stabilization
1	21/M	310	Dive	C5	+	+	Halo vest
2	34/M	482	Traffic accident	C5	–	–	Halo vest
3	28/M	867	Traffic accident	C5	+	+	Neck brace
4	15/M	1874	Dive	C6	+	–	Halo vest
5	14/M	1144	Dive	C5	+	–	Halo vest
6	17/M	3451	Dive	C5	+	–	Neck brace
7	41/M	726	Fall	C6	–	+	Neck brace
8	30/M	969	Dive	C6	–	–	Halo vest
9	32/M	282	Traffic accident	C6	–	–	Neck brace
10	68/M	1823	Traffic accident	C7	–	–	Neck brace
11	19/M	411	Traffic accident	C5	–	+	Neck brace
12	22/M	1110	Traffic accident	C4	+	+	Neck brace
13	28/M	715	Fall	C5	+	+	Halo vest

**Table 2:** Neurological status of American Spinal Injury Association (ASIA) Impairment Scale before and after injury

Case	AIS at the time of injury	AIS at the time of final follow-up
1	B	B
2	D	E
3	B	D
4	B	B
5	C	D
6	C	D
7	A	A
8	D	E
9	D	E
10	D	E
11	D	E
12	D	D
13	D	E

**Table 3:**
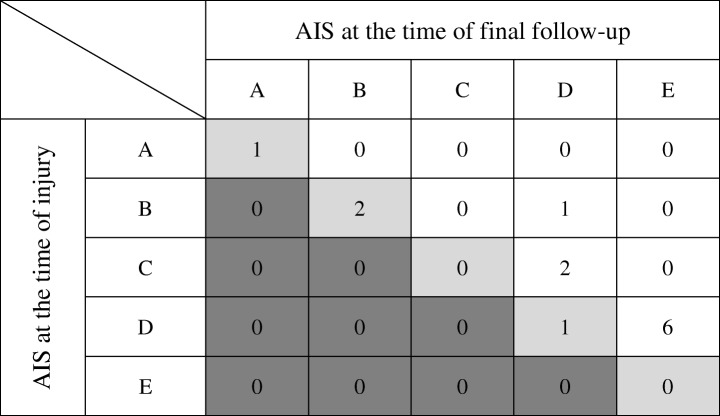
Diagram showing change in ASIA Impairment Scale between the status at the time of injury (vertical axis) and at the time of final follow-up (horizontal axis)

**Fig. 2:**
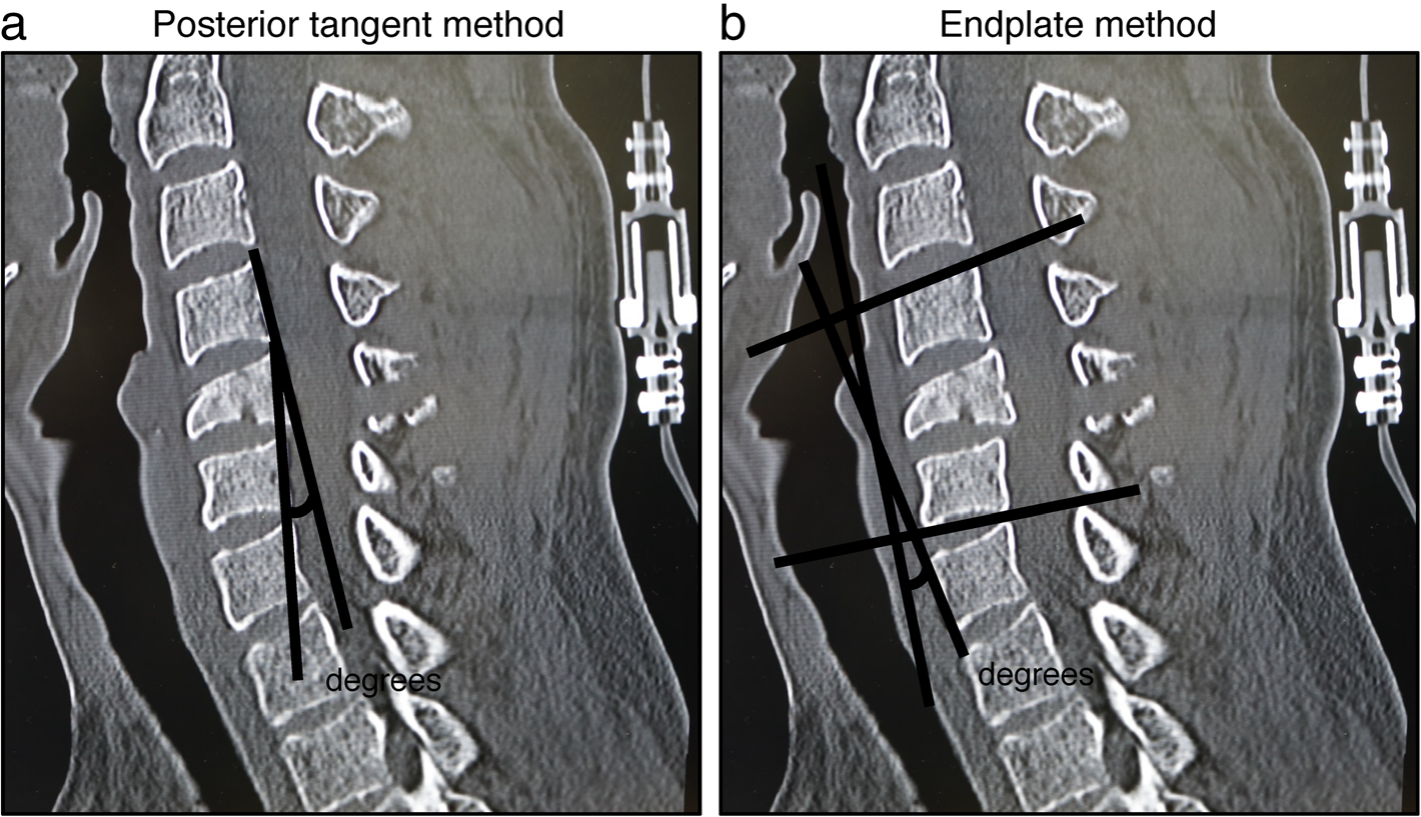
Radiographic measurements for local cervical kyphosis. **a** Diagram depicting the measurement technique for local kyphosis using the posterior tangent method. The angle between the lines drawn along the posterior vertebral body was measured. **b** Diagram depicting the measurement technique for local kyphosis using the endplate method. The angle of intersection between a line drawn along the superior endplate of the suprajacent vertebra and a line drawn along the inferior endplate of the infrajacent vertebra was measured

**Fig. 3:**
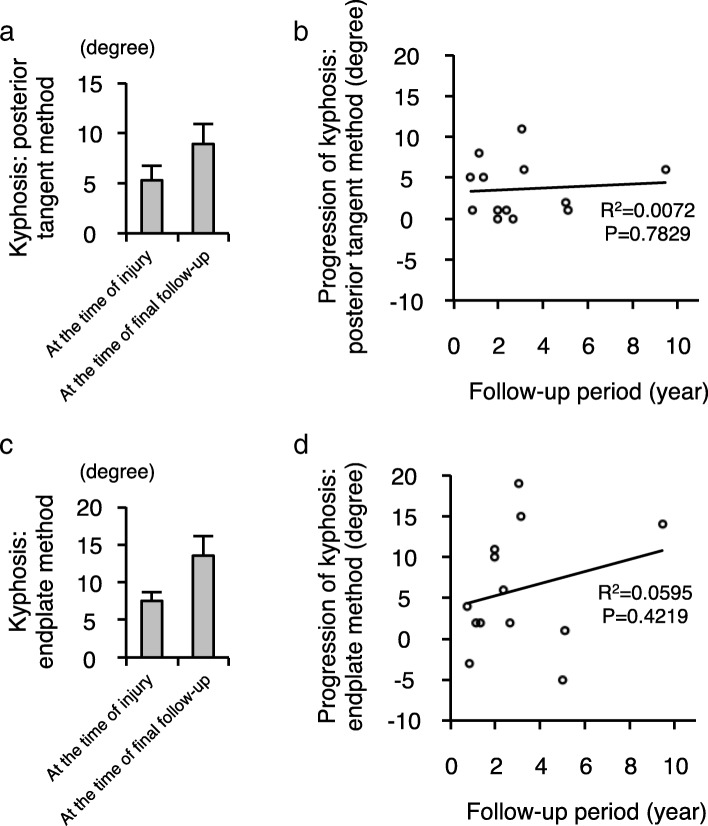
The overall progression of local kyphosis after compressive cervical spine fractures. **a** The results of the local kyphosis at the time of injury (*n* = 13) and at the time of final follow-up (*n* = 13) measured by the posterior tangent method. **b** The relationship between the progression of local kyphosis measured by the posterior tangent method and the duration of the follow-up period. **c** The results of the local kyphosis at the time of injury (*n* = 13) and at the time of final follow-up (*n* = 13) measured by the endplate method. **d** The relationship between the progression of local kyphosis measured by the endplate method and the duration of the follow-up period. There is little correlation between the progression of local cervical kyphosis and the duration of the follow-up period

**Fig. 4:**
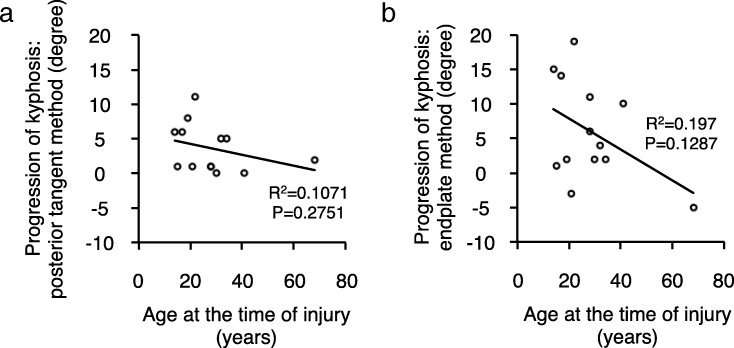
Moderate negative correlation between the age at the time of injury and the progression of local kyphosis. **a** The relationship between the progression of local kyphosis measured by the posterior tangent method and the age at the time of injury. **b** The relationship between the progression of local kyphosis measured by the endplate method and the age at the time of injury. Moderate negative correlation was found between the progression of kyphosis and the age at the time of injury

To evaluate local kyphosis of the cervical spine, we applied two radiographic methods: the posterior tangent method and the endplate method (Fig. 2) [19]. We found overall progression of kyphosis at the time of final follow-up compared to that was observed at the time of injury using both methods (Fig. 3). However, there was no significant relationship between the length of the follow-up period and the progression of the local kyphosis (Pearson rank correlation coefficient: progression of kyphosis vs. follow-up period, posterior tangent method, *R*^2^ = 0.0072, *P* = 0.7829; endplate method, *R*^2^ = 0.0595, *P* = 0.4219) (Fig. 3). Interestingly, we observed a moderate negative correlation between the age at the time of injury and the progression of the local kyphosis (progression of kyphosis vs. age at the time of injury, posterior tangent method, *R*^2^ = 0.1071, *P* = 0.2751; endplate method, *R*^2^ = 0.197, *P* = 0.1287) (Fig. 4).

**Fig. 5:**
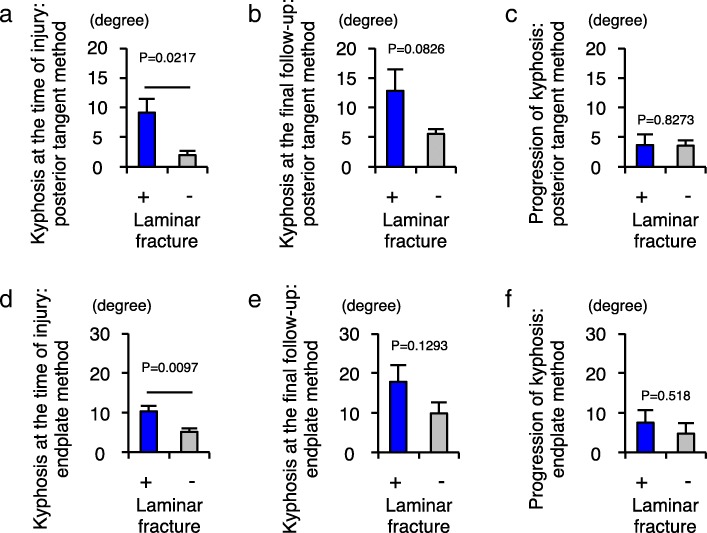
Laminar fractures affect the initial kyphotic alignment after injury and do not affect the progression of local kyphosis. **a** Comparison of local cervical kyphosis between the two groups of patients with (*n* = 6) and without (*n* = 7) laminar fracture, as measured by the posterior tangent method at the time of injury. **b** Comparison of local cervical kyphosis between the two groups of patients with and without laminar fracture, as measured by the posterior tangent method at the time of final follow-up. **c** Comparison of the progression of local cervical kyphosis between the two groups of patients with and without laminar fracture as measured by the posterior tangent method. **d** Comparison of the local cervical kyphosis between the two groups of patients with and without laminar fracture as measured by the endplate method at the time of injury. **e** Comparison of local cervical kyphosis between the two groups of patients with and without laminar fracture as measured by the endplate method at the time of final follow-up. **f** Comparison of the progression of local cervical kyphosis between the two groups of patients with and without laminar fracture as measured by the endplate method

We next examined whether laminar fracture or vertical fracture of the vertebral column affected the progression of local kyphosis, since these two types of fractures have been reported to be influencing factors on spinal alignment [20, 21]. We found a significantly higher degree of local kyphosis at the time of injury in the group with laminar fracture compared to that in the group without laminar fracture (posterior tangent method, *P* = 0.0217; endplate method, *P* = 0.0097). However, we observed no significant difference in the progression of local kyphosis between the group with laminar fracture and the group without laminar fracture (posterior tangent method, *P* = 0.8273; endplate method, *P* = 0.518) (Fig. 5). These findings suggest that posterior column fractures affect the initial kyphotic alignment after injury and do not affect the progression of local kyphosis over the longer term.

**Fig. 6:**
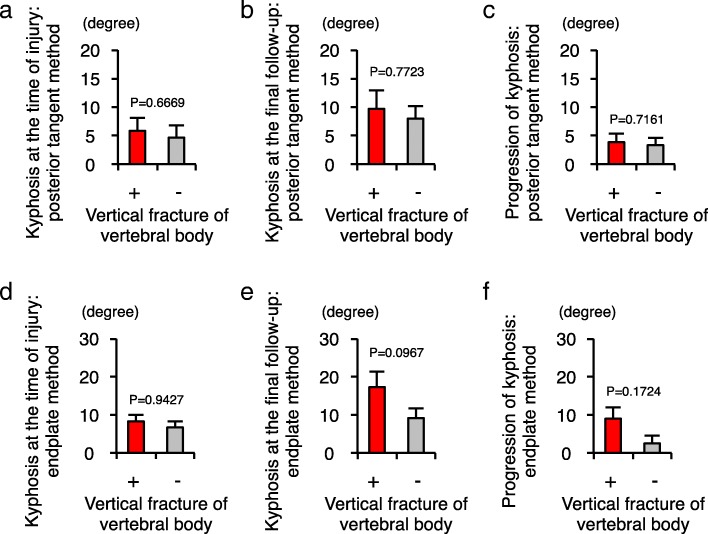
Vertical fracture of the vertebral body could be an influencing factor for the progression of local kyphosis. **a** Comparison of the local cervical kyphosis between the two groups of patients with (*n* = 7) and without (*n* = 6) vertical fracture of the vertebral body, as measured by the posterior tangent method at the time of injury. **b** Comparison of the local cervical kyphosis between the two groups of patients with and without vertical fracture of the vertebral body as measured by the posterior tangent method at the time of final follow-up. **c** Comparison of the progression of local cervical kyphosis between the two groups of patients with and without vertical fracture of the vertebral body as measured by the posterior tangent method. **d** Comparison of the local cervical kyphosis between the two groups of patients with and without vertical fracture of the vertebral body as measured by the endplate method at the time of injury. **e** Comparison of the local cervical kyphosis between the two groups of patients with and without vertical fracture of the vertebral body as measured by the endplate method at the time of final follow-up. **f** Comparison of the progression of local cervical kyphosis between the two groups of patients with and without vertical fracture of the vertebral body as measured by the endplate method

**Fig. 7:**
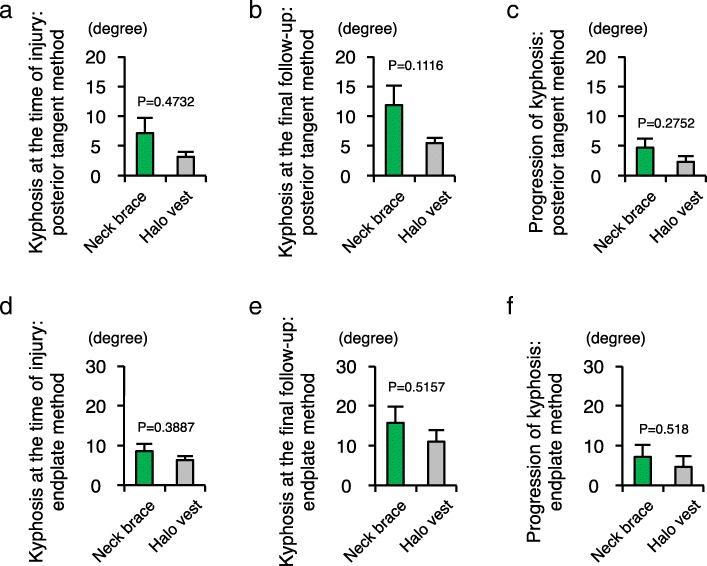
No significant difference in the progression of local kyphosis between the two orthosis treatments. **a** Comparison of the local cervical kyphosis between the group of patients treated with neck brace (*n* = 7) and the group of patients treated with halo vest (*n* = 6) as measured by the posterior tangent method at the time of injury. **b** Comparison of the local cervical kyphosis between the group of patients treated with neck brace and the group of patients treated with halo vest as measured by the posterior tangent method at the time of final follow-up. **c** Comparison of the progression of local cervical kyphosis between the group of patients treated with neck brace and the group of patients treated with halo vest as measured by the posterior tangent method. **d** Comparison of the local cervical kyphosis between the group of patients treated with neck brace and the group of patients treated with halo vest as measured by the endplate method at the time of injury. **e** Comparison of the local cervical kyphosis between the group of patients treated with neck brace and the group of patients treated with halo vest as measured by endplate method at the time of final follow-up. **f** Comparison of the local cervical kyphosis between the group of patients treated with neck brace and the group of patients treated with halo vest as measured by the endplate method

With regard to the influence of vertebral vertical fractures, the degrees of local kyphosis at the time of injury were comparable between the groups with and without vertical fractures (posterior tangent method, *P* = 0.6669; endplate method, *P* = 0.9427). As measured by the endplate method, the progression of kyphosis in the group with vertical fracture was higher than in the group without vertical fracture, but this difference was not significant (*P* = 0.1724) (Fig. 6). We further examined whether the type of orthosis as a conservative therapy affected the progression of kyphosis after injury. There was no significant difference in the progression of kyphosis between the group treated by neck brace and the group treated by halo vest (posterior tangent method, *P* = 0.2752; endplate method, *P* = 0.518) (Fig. 7).

### Representative case

**Fig. 8:**
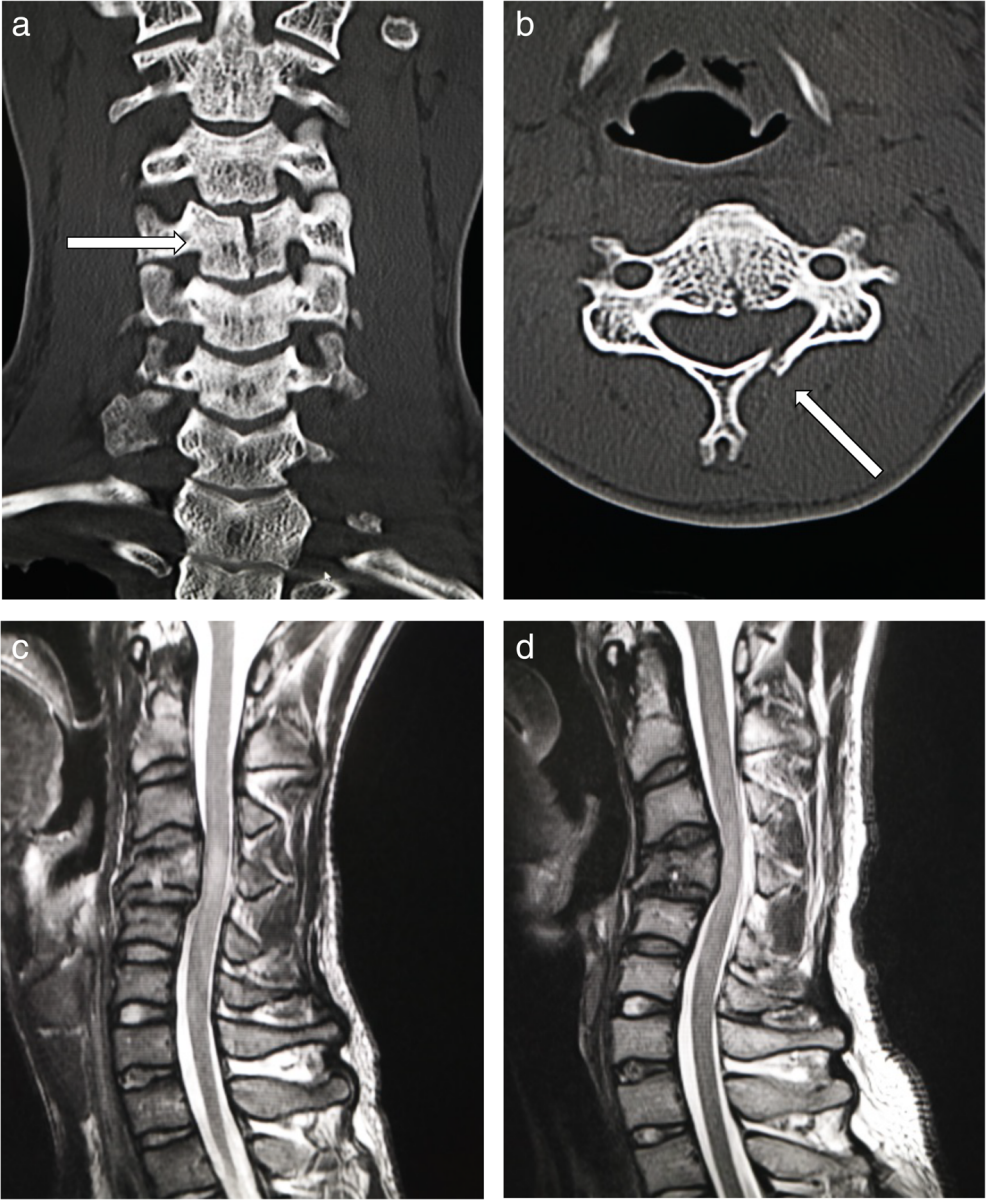
A representative case with progression of local kyphosis. **a**, **b** Computed tomography (CT) images at the time of injury showed the vertebral vertical fracture and left laminar fracture at the C4 level. **c** Cervical spinal cord injury was confirmed by T2-weighted magnetic resonance imaging (MRI) at the time of injury. **d** MRI at the time of final follow-up (1110 days after injury) showed the progression of local cervical kyphosis from 17 to 36°, measured by the endplate method. His neurological finding remained at AIS grade D and improved to modified Frankel grade D3

A 22-year-old man had been hit while walking by a heavy-duty, eight-ton vehicle. The ASIA Impairment Scale (AIS) at the time of admission was diagnosed as grade D. Also, in terms of the modified Frankel grading system [22, 23], he was diagnosed as grade D0. The manual muscle test scores (MMTs) of the lower extremities were 4–5, and we were unable to test his walking ability at the time of his hospitalization. MRI and CT images showed a compression fracture at C4, with both vertebral vertical fracture and laminar fracture (Fig. 8). Intramedullary T2-weighted signal change was observed at the C4 level. He was treated conservatively with a rigid neck brace and intensive rehabilitation due to his multiple traumatic injuries, such as subdural hygroma, radius shaft fracture, tibial shaft fracture, and fibula shaft fracture. At the time of final follow-up, approximately 3 years after the injury, while his neurological function remained at AIS grade D, his Frankel grade had improved to D3. His gait was independent and without use of a cane. However, his local kyphosis had progressed from 18 to 36°, as measured by the endplate method, and from 11 to 28°, as measured by the posterior tangent method, whereas the bony fusion had been completed (Fig. 8).

## Discussion

We presented several significant findings in this study. First, local kyphosis progressed after conservative treatment for patients with compressive cervical spine fractures with spinal cord injury. Second, the age at the time of injury and the vertical fracture of the vertebral body were influencing factors on the progression of local kyphosis, while the period of follow-up, presence of laminar fractures, and the type of orthosis were not. Third, conservative treatment of patients with compressive cervical spine fracture with spinal cord injury did not exacerbate neurological dysfunction or lead to non-union of the fracture. From the viewpoint of cervical alignment, younger patients and patients with vertical vertebral fractures demonstrated a higher incidence of local kyphosis progression, and spinal surgeons should be aware that this type of injury may lead to cervical deformity.

We used two means of evaluating local kyphosis: the endplate method and the posterior tangent method. From the viewpoint of the endplate method, vertical fracture of the vertebral body was a risk factor for the progression of local kyphosis in terms of long-term outcome. Conversely, the posterior tangent method did not prove this relationship, as shown in Fig. 6. Compressive-flexion fracture of the cervical spine leads to the loss of vertebral height over the longer term, and the injured vertebral bone becomes wedge-shaped [21, 24]. Indeed, Bono and colleagues suggested that the posterior tangent method is preferred for evaluating kyphosis in patients with non-traumatic pathology, such as degenerative cervical spines [19], which supports the notion that the posterior tangent method cannot sufficiently detect vertebral vertical fracture as a risk factor for the progression of local kyphosis. Although both measurement methods are straightforward and useful for assessing the local kyphosis, the endplate method appears to be the more reasonable method for evaluating kyphosis, especially in patients with vertebral fractures.

It remains controversial whether any local kyphosis of the cervical spine is acceptable after spinal cord injury [25]. Jenkins and colleagues previously showed that patients with cervical kyphosis of 20° or more had severe cervical pain symptoms [24]. They also demonstrated that the pain symptoms did not correlate with instability below the fusion level or disc space narrowing. Although there were five patients (38%) with cervical kyphosis of more than 20° in the present study, none of these patients complained of any cervical pain, which can possibly be attributed to the fact that the bilateral facet joints of these patients were preserved. Future studies investigating the link between cervical kyphosis and symptoms, such as severe neck pain, that affect activities of daily life could provide additional information establishing evidence for a surgical or non-surgical approach after cervical spinal fracture with spinal cord injury.

The nationwide databases showed that the incidence of cervical fractures with or without spinal cord injury among the elderly population beyond 65 years is increasing over the past decade. Most patients included in this population group have multiple comorbidities such as cancer, heart failure, cerebrovascular disease, and diabetes, and are receiving treatments for these conditions before sustaining an injury [26, 27]. Adjusted-population rates of in-hospital mortality after spinal injury also revealed increasing trends in the elderly [28]. Furthermore, hospitalization costs of spinal cord injury patients with cervical spine fractures are increasing, with greater costs being incurred from the increase in surgeries. Given that this study shows that conservative treatment is feasible for patients with this spine fracture, the need to study the changes in cervical alignment after conservative treatment of older patients is apparent. Since our current patient population did not have sufficient numbers of elderly cases, we hope to analyze and report on this point in the future when we accumulate sufficient cases.

There are several potential limitations associated with our study. First, this retrospective study was extremely limited in size. A larger sample size is required to elucidate the impact of vertical fracture of the cervical spine and even laminar fracture on the progression of local kyphosis. Second, we did not compare this conservatively treated group with the group treated by surgery, such as cervical decompression and fixation. A prospective and randomized study is needed to confirm the preventive effect on local kyphosis by surgery. We encountered very few patients with compressive cervical fracture whose facet joints had maintained their congruity. Moreover, on the basis of previous studies, most patients with this type of injury may have to be treated by surgery [13]. Considering that these patients typically suffer high-energy trauma with concurrent injury in other critical organs, we believe that our study is essential in terms of understanding the effectiveness of conservative treatment for patients with both cervical spine fracture and spinal cord injury.

## Conclusion

We presented cases that had progression of local cervical kyphosis after conservative treatment for compressive-flexion cervical fractures with spinal cord injury. Even though conservative treatment of this injury was possible without exacerbating neurological symptoms, younger patients and patients with vertical vertebral fractures demonstrated a higher incidence of local kyphosis progression.

## Abbreviations

AIS: ASIA Impairment Scale
ASIA: American Spinal Injury Association
CT: Computed tomography
MMTs: Manual muscle test scores
MRI: Magnetic resonance imaging

## References

[1] Pickett GE, Campos-Benitez M, Keller JL, Duggal N (2006). Epidemiology of traumatic spinal cord injury in Canada. Spine (Phila Pa 1976).

[2] Allen BL, Ferguson RL, Lehmann TR, O’Brien RP (1982). A mechanistic classification of closed, indirect fractures and dislocations of the lower cervical spine. Spine (Phila Pa 1976).

[3] Raniga SB, Menon V, Al Muzahmi KS, Butt S (2014). MDCT of acute subaxial cervical spine trauma: a mechanism-based approach. Insights Imaging.

[4] Vaccaro AR, Koerner JD, Radcliff KE, Oner FC, Reinhold M, Schnake KJ, Kandziora F, Fehlings MG, Dvorak MF, Aarabi B (2016). AOSpine subaxial cervical spine injury classification system. Eur Spine J.

[5] Dogan S, Safavi-Abbasi S, Theodore N, Horn E, Rekate HL, Sonntag VK (2006). Pediatric subaxial cervical spine injuries: origins, management, and outcome in 51 patients. Neurosurg Focus.

[6] Krueger H, Noonan VK, Trenaman LM, Joshi P, Rivers CS (2013). The economic burden of traumatic spinal cord injury in Canada. Chronic Dis Inj Can.

[7] Coleman JA, Harper LA, Perrin PB, Olivera SL, Perdomo JL, Arango JA, Arango-Lasprilla JC (2015). The relationship between physical and mental health variables in individuals with spinal cord injury from Latin America. Pm r.

[8] Lee AS, Wainwright AM, Newton DA (1996). Rogers’ posterior cervical fusion—a 3-month radiological review. Injury.

[9] Kim HJ, Lee KY, Kim WC (2009). Treatment outcome of cervical tear drop fracture. Asian Spine J.

[10] Favero KJ, Van Peteghem PK. The quadrangular fragment fracture. Roentgenographic features and treatment protocol. Clin Orthop Relat Res. 1989;239:40–6. 2912635

[11] Yue JK, Winkler EA, Rick JW, Deng H, Partow CP, Upadhyayula PS, Birk HS, Chan AK, Dhall SS (2017). Update on critical care for acute spinal cord injury in the setting of polytrauma. Neurosurg Focus.

[12] Dvorak MF, Fisher CG, Fehlings MG, Rampersaud YR, Oner FC, Aarabi B, Vaccaro AR (2007). The surgical approach to subaxial cervical spine injuries: an evidence-based algorithm based on the SLIC classification system. Spine (Phila Pa 1976).

[13] Vaccaro AR, Hulbert RJ, Patel AA, Fisher C, Dvorak M, Lehman RA, Anderson P, Harrop J, Oner FC, Arnold P (2007). The subaxial cervical spine injury classification system: a novel approach to recognize the importance of morphology, neurology, and integrity of the disco-ligamentous complex. Spine (Phila Pa 1976).

[14] Iida H, Tachibana S, Kitahara T, Horiike S, Ohwada T, Fujii K (1999). Association of head trauma with cervical spine injury, spinal cord injury, or both. J Trauma.

[15] Markandaya Manjunath, Stein Deborah M., Menaker Jay (2012). Acute Treatment Options for Spinal Cord Injury. Current Treatment Options in Neurology.

[16] Hagen EM (2015). Acute complications of spinal cord injuries. World J Orthop.

[17] Bono CM, Schoenfeld A, Gupta G, Harrop JS, Anderson P, Patel AA, Dimar J, Aarabi B, Dailey A, Vaccaro AR (2011). Reliability and reproducibility of subaxial cervical injury description system: a standardized nomenclature schema. Spine (Phila Pa 1976).

[18] Kirshblum SC, Burns SP, Biering-Sorensen F, Donovan W, Graves DE, Jha A, Johansen M, Jones L, Krassioukov A, Mulcahey MJ (2011). International standards for neurological classification of spinal cord injury (revised 2011). J Spinal Cord Med.

[19] Bono CM, Schoenfeld A, Rampersaud R, Levi A, Grauer J, Arnold P, Fehlings M, Dvorak M, Vaccaro AR (2011). Reproducibility of radiographic measurements for subaxial cervical spine trauma. Spine (Phila Pa 1976).

[20] Okamoto Y, Murakami H, Demura S, Kato S, Yoshioka K, Hayashi H, Sakamoto J, Kawahara N, Tsuchiya H (2015). The effect of kyphotic deformity because of vertebral fracture: a finite element analysis of a 10 degrees and 20 degrees wedge-shaped vertebral fracture model. Spine J.

[21] Kim KS, Chen HH, Russell EJ, Rogers LF (1989). Flexion teardrop fracture of the cervical spine: radiographic characteristics. AJR Am J Roentgenol.

[22] Frankel HL, Hancock DO, Hyslop G, Melzak J, Michaelis LS, Ungar GH, Vernon JD, Walsh JJ (1969). The value of postural reduction in the initial management of closed injuries of the spine with paraplegia and tetraplegia. I. Paraplegia.

[23] Hayashi T, Kawano O, Sakai H, Ideta R, Ueta T, Maeda T, Mori E, Yugue I, Takao T, Masuda M (2013). The potential for functional recovery of upper extremity function following cervical spinal cord injury without major bone injury. Spinal Cord.

[24] Jenkins LA, Capen DA, Zigler JE, Nelson RW, Nagelberg S. Cervical spine fusions for trauma. A long-term radiographic and clinical evaluation. Orthop Rev. 1994;(Suppl):13–9. 7854834

[25] Kim SW, Kim TH, Bok DH, Jang C, Yang MH, Lee S, Yoo JH, Kwak YH, Oh JK (2018). Analysis of cervical spine alignment in currently asymptomatic individuals: prevalence of kyphotic posture and its relationship with other spinopelvic parameters. Spine J.

[26] Asemota AO, Ahmed AK, Purvis TE, Passias PG, Goodwin CR, Sciubba DM (2018). Analysis of cervical spine injuries in elderly patients from 2001 to 2010 using a nationwide database: increasing incidence, overall mortality, and inpatient hospital charges. World Neurosurg.

[27] Krassioukov AV, Furlan JC, Fehlings MG (2003). Medical co-morbidities, secondary complications, and mortality in elderly with acute spinal cord injury. J Neurotrauma.

[28] Golob JF, Claridge JA, Yowler CJ, Como JJ, Peerless JR (2008). Isolated cervical spine fractures in the elderly: a deadly injury. J Trauma.

